# Antimicrobial Activity of Silver Containing Crosslinked Poly(Acrylic Acid) Fibers

**DOI:** 10.3390/mi10120829

**Published:** 2019-11-28

**Authors:** Mohammad Mofidfar, Eun Seon Kim, Emily L. Larkin, Lisa Long, Wayne D. Jennings, Samad Ahadian, Mahmoud A. Ghannoum, Gary E. Wnek

**Affiliations:** 1 Department of Macromolecular Science and Engineering, Case Western Reserve University, Cleveland, OH 44106, USA; kes1207@gmail.com; 2 Department of Dermatology and Center for Medical Mycology, Case Western Reserve University, and University Hospitals Case Medical Center, Cleveland, OH 44106, USA; ell33@case.edu (E.L.L.); Lal11@cwru.edu (L.L.); mag3@case.edu (M.A.G.); 3 Swagelok Center for Surface Analysis of Materials, Case Western Reserve University, Cleveland, OH 44106, USA; wdj@case.edu; 4 Department of Bioengineering, Henry Samueli School of Engineering and Applied Sciences, University of California-Los Angeles, Los Angeles, CA 90095, USA; smd.ahadian@gmail.com; 5 Center for Minimally Invasive Therapeutics (C-MIT), University of California-Los Angeles, Los Angeles, CA 90095, USA

**Keywords:** antimicrobial activity, silver nanoparticles, poly(acrylic acid) fibers, electrospinning

## Abstract

Bacterial and fungal pathogens have caused serious problems to the human health. This is particularly true for untreatable infectious diseases and clinical situations where there is no reliable treatment for infected patients. To increase the antimicrobial activity of materials, we introduce silver nanoparticle (NP) patches in which the NPs are incorporated to the surface of smooth and uniform poly(acrylic acid) (PAA) nanofibers. The PAA nanofibers were thermally crosslinked with ethylene glycol via heat treatment through a mild method. The characterization of the resulting PAA-silver NP patches was done using scanning electron microscopy (SEM), UV spectroscopy, X-ray diffraction (XRD), and X-ray photoelectron spectroscopy (XPS). To demonstrate the antimicrobial activity of PAA, we incorporated the patches containing the silver NPs into strains of fungi such as *Candida albicans* (*C. albican*) and bacteria such as Methicillin-resistant *Staphylococcus aureus* (MRSA). The PAA-silver fibers achieved zones of inhibition against *C. albicans* and MRSA indicating their antimicrobial activity against both fungi and bacteria. We conclude that silver NP patches exhibited multiple inhibitory actions for the interruption and blockage of activity fungal and bacterial strains, which has the potential as an antimicrobial agent in infectious diseases. Moreover, the proposed material has the potential to be used in antimicrobial textile fabrics, food packaging films, and wound dressings.

## 1. Introduction 

Resistance among common bacterial and fungal pathogens due to excessive use of antibiotics makes them a serious problem to the human health. In the United States, at least 23,000 deaths have been reported annually due to infection with an antibiotic-resistant organism [1,2]. Antibiotic resistance is a major public health threat in our century according to the World Health Organization report [3]. For example, a recent study found that antibiotic resistance could cause at least 300 million premature deaths worldwide causing up to $100 trillion loss to the world economy by 2050 [4]. This situation requires a robust antibiotic pipeline as a pathway to bring new devices and medicines, focusing on untreatable infectious diseases and supporting patients and clinicians with no reliable alternatives to treat infected patients.

To address this major challenge, novel nanosized platforms and antimicrobial nanomaterials have been proposed where microbial pathogens cannot develop effective resistance [5,6]. Recent studies have found that resistance to antibiotics and antifungals has been rapidly increasing to a critical level, which limits the usage of current drugs in hospitals and communities [3]. However, current progress in the development of synthetic drugs has been very slow [7]. Despite the current progress in the preparation of conventional antibiotics, these methods may be costly and time-consuming and use biohazardous materials [8].

Some chemical, physical, and biological methods have been reported as excellent methods to prepare antimicrobial techniques and thereby they have been used for loading silver NPs on fiber patches. These fibrous patches are defined as a part of fabric or a material over a hole or weak point in the patches to deliver a specific dose of medication through the skin. Commercial ability to produce silver nanoparticles (NPs) has led to many biomedical applications of these NPs, such as antifouling hydrogels [9], antimicrobial activities (microorganism reduction) [10,11], eliminating toxic chemicals in water, as well as treatment of wounds and burns [12]. Kim et al. used poly(vinyl alcohol)/Ag-zeolite nanofibers for an antibacterial efficacy against Klebsiella pneumoniae and *Staphylococcus aureus* due to small sizes and relative surface area of nanofibers in contact with the outer membrane of target cells [13]. Xu et al. obtained poly(L-lactide)/Ag fibers through addition of different concentrations of AgNO_3_ in polylactic acid solution and then the reduction of silver nitrate at 80 °C [9]. In addition, most studies incorporated Ag NPs to electrospun nanofibers through mixing of antibacterial agents in the electrospinning solution [14,15,16,17]. Using these methods, silver NPs can be encapsulated within or on the surface of nanofibers. 

A limitation of synthetic NP development is that they need harsh conditions, such as high temperature sterilization compared to preparation of conventional antibiotics. While high solubility and cellular internalization of antibacterial agents have been achieved, homogeneous dispersion of NPs on the surface of polymeric matrix is considered an important challenge [18,19,20]. An expectation of novel approaches to synthesize NPs for biomedical applications is their green chemistry through mild reaction conditions, which do not involve toxic solvents and ingredients [21]. 

Here, we propose the use of poly(acrylic acid) (PAA) nanofibers to incorporate silver NPs. We developed silver NPs incorporated into the crosslinked PAA fiber patches and then studied their antifungal activity against *C. albicans* and their antibacterial activity against Methicillin-resistant *Staphylococcus aureus* (MRSA). This approach may be appealing because silver NP-loaded patches are inexpensive, and more importantly, are active against both fungi and bacteria. We also report the characterization of silver NP-loaded fiber patches by SEM, XRD and XPS, and in vitro characterization of antimicrobial activity of the patches. 

## 2. Experimental Section

### 2.1. Materials

**Figure 1:**
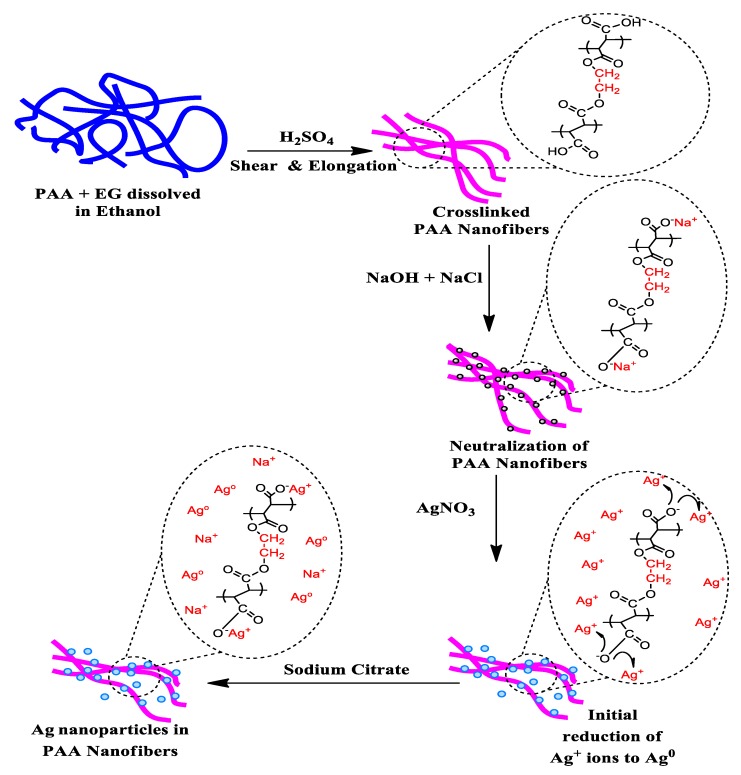
Illustration of Ag nanoparticles (NPs) incorporation in poly(acrylic acid) (PAA) nanofibers.

PAA (average M_w_ = 450,000 g/mol), ethylene glycol (EG, anhydrous), silver nitrate (AgNO_3_), and sodium citrate were supplied by Sigma-Aldrich (St. Louis, MO, USA) and used without purification. Sodium hydroxide (NaOH) and sodium chloride (NaCl) were purchased from Fisher Scientific (Fair Lawn, NJ, USA) and used as received. The following strains were obtained from Case Western Reserve University Center for Medical Mycology (Cleveland, OH, USA) and used as the test organisms; *C. albicans* SC5314, and MRSA USA 300. The preparation of silver NPs was done in 4 steps as detailed below (Figure 1). 

### 2.2. Methods 

#### 2.2.1. Preparation of PAA Ethanol Solution, PAA Nanofibers, and Thermal Crosslinking of Electrospun PAA Nanofiber (Step I)

PAA solution (4 wt%) was made as a result of dissolving PAA in ethanol. EG was then added as a crosslink agent at the concentration 16 wt% relative to the polymer. The PAA and EG were dissolved in ethanol and stirred for 24 h at 25 °C to obtain a homogenous solution. Before electrospinning, 1 M sulfuric acid as a catalyst was mixed with the PAA–EG solution (50 µL/mL) for thermal esterification. Sulfur is a good disinfectant and a toxic composition, but the addition of a small amount of sulfuric acid enhances the stability of Ag ions. 

PAA nanofibers were fabricated via optimal electrospinning conditions by PAA–EG solution that was fed through the 18 gauge hypodermic needle tip at a voltage of 15 kV and flow rate of 0.8 mL/h. A rotating collection target was placed 20 cm away from the needle tip. Thermal crosslinking conditions of the PAA nanofibers included the heat treatment at 130 °C under vacuum (−25 in Hg) for 30 min, and then cooled down to ambient temperature. The heat treatment caused the formation of intermolecular crosslinking between the –COOH groups of the PAA and the –OH groups of the EG. 

#### 2.2.2. Neutralization of PAA Nanofibers (Step II)

PAA nanofibers in the carboxylic acid form (PAA–H) were transformed into the ones in sodium carboxylate form (PAA–Na) by swelling in a mixture solution of 1 M NaOH and 1 M NaCl for approximately 1 h. Residual salts in nanofibers were removed through rinsing with water. After neutralization, the uncrosslinked carboxylic acid (–COOH) groups of the PAA were changed to sodium carboxylates (–Na^+^ ions).

#### 2.2.3. Preparation of PAA Nanofibers with Silver Ions (Step III)

PAA–Na fibers were soaked in 20 mL of AgNO_3_ (5 mM) for approximately 90 min and then placed in water to remove residual salts. This step can replace sodium ions (–Na^+^) in the PAA nanofibers with silver ions (–Ag^+^). 

#### 2.2.4. Preparation of Hybrid of Silver-PAA Nanofibers (Step IV) 

PAA nanofibers with silver ions were immersed in 20 mL of 25 mM sodium citrate solution during 4 d and then placed in water to remove residual salts. Sodium citrate was used as a reduction agent for silver ions in the PAA nanofibers. The silver NP-containing PAA fibers were removed from the solution and kept in deionized water for future use. 

### 2.3. Characterization 

Morphology of prepared PAA nanofibers was assessed using high resolution field emission SEM (FEI-Nova NanoLab 200 FEG-SEM/FIB, Hillsboro, OR, Country), a current of 1 nA, and an acceleration voltage of 5 kV. For SEM measurements, 10 nm gold was sputter-coated on electrospun fibers. Energy dispersive X-ray spectroscopy (EDS, Oxford Instruments plc, Tubney Woods, UK) was used for the elemental analysis by the aid of an OXFORD X-Max with a 50 mm^2^ silicon drift detector. The XRD spectra were obtained using a Bruker Discover D8 (Bruker, Karlsruhe, Germany) with Co Kα radiation (1.79026 Å) and X-ray beam diameter of 500 μm. A Shimadzu UV-1800 spectrophotometer in a wavelength range between 190–700 nm was used to collect UV-Vis spectra. Chemical elements on PAA nanofibers were specified using XPS using a PHI 5600 ESCA system (Ulvac-Phi Inc., Kanagawa, Japan). For the XPS measurements, high-resolution scans (0.1 eV step) and 20–60 min survey scans in 0.4 eV step were done in the binding energy range of 0–1100 eV in areas with a monochromatic Al K-alpha X-ray. 

### 2.4. Antifungal and Antimicrobial Testing

The antibacterial and antifungal activities of PAA nanofibers with silver NPs were evaluated from the culture collection against (fungi) *C. albicans* SC5314 and (bacteria) MRSA USA 300 using disk diffusion. In these instances, the cultures were filled with PAA-silver fibers and then incubated for up to 24 h at 37 °C, and the diameter of inhibition zone was measured to determine around the tested samples. The antibacterial activities of PAA nanofibers with and without silver ions and particles were evaluated against MRSA by disk diffusion method to measure the diameter of inhibition zone under and around the tested samples. Brain heart infusion agar was autoclaved at 120 °C for 15 min and cooled in sterile Petri-dishes to from a 2 mm thick slab. For antibacterial assessment, 300 mL of bacterial solution with about 10^8^ CFU·mL^−1^ of MRSA was dispensed onto an agar plate and then hybrid PAA-silver nanofibers samples were covered on the surface of the plate. For antifungal assessment, 300 mL of *C. albican* solution with about 3–5 × 10^7^ CFU·mL^−1^ was covered onto the agar and plate. Then PAA-silver containing nanofibers (5 mM AgNO_3_) were placed onto the agar surface. After incubation at 37 °C for 24 h, the bacterial growth inhibition halos were observed.

## 3. Results and Discussion 

Silver salt reduction by sodium borohydride and sodium citrate is the most popular preparation method of Ag colloids. Here, the reduction of silver NPs in PAA was carried out by sodium citrate reduction method. Sodium citrate is a weak reductant compared to sodium borohydride, hydrazine, and hydroquinone and has strong surface interaction as exposed to silver nanocrystallites. Complexation of citrate and colloidal silver causes slow crystal growth in contrast to other radiolytic and chemical approaches [22,23]. Since this reduction process can control the growth rate through the dual rule of citrate and strong surface interaction, it is possible to control the shape and size of the silver NPs. It was obvious that silver NPs aggregate on the surface of PAA nanofibers during deposition. The reduction reaction silver cations in sodium citrate can be described by Equation (1):

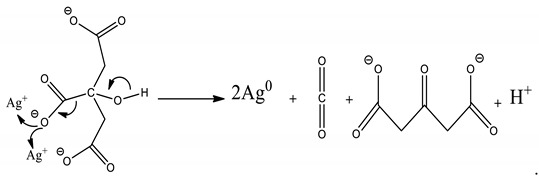
(1)

Stable and reproducible colloids can be produced through the chemical reduction of silver salts by sodium citrate. The purity of water and reagents are important factors in the reduction process. Furthermore, the agglomeration of silver atoms forms silver nanoclusters that can grow into the large clusters described by Equation (2):

n Ag^0^ → (Ag)_n_. (2)

The addition of AgNO_3_ solution to the polyanionic hydrogel nanofiber causes bounding of Ag^+^ ions to acrylic acid groups through electrostatic interaction between the oxygen atoms of either the hydroxyl or the carbonyl groups. 

### 3.1. Morphology and Structural Characterization

**Figure 2:**
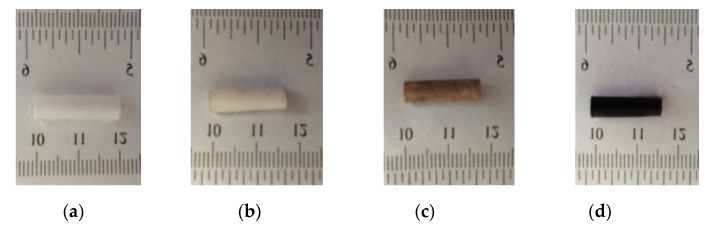
The schematic representing the mechanism for the formation of silver nanoparticles and color change in PAA tubes at difference steps (**a**) electrospun PAA nanofiber, (**b**) neutralization of PAA nanofibers, (**c**) PAA nanofibers with silver ions (brown color), and (**d**) hybrid of PAA-silver nanofibers (dark color).

Figure 2 describes the physical appearance of silver NPs within the electrospun hydrogel network during the process. The cylindrical morphology of continuous PAA electrospun nanofibers was obtained by rotating a cylindrical collector at a very high speed of up to thousands of rpm. Reduction and/or nucleation rates in the formation of Ag NPs are slower as evidenced by the delayed appearance of the characteristic yellow color. This process turns the color slightly brown due to the presence of Ag NPs. The colloidal solution gradually changed its color during the reaction, which confirmed the Ag^+^ reduction and the formation of silver NPs on the electrospun hydrogel network. The most notable feature of our process is the formation of highly stable silver NPs embedded on the carboxylate group of the PAA fiber network. The untreated PAA nanofibers were milky white as shown in Figure 2a. The color of PAA nanofibers became brown after addition of AgNO_3_ solution because silver exists in the form of Ag^+^ ions. The mechanism with which the PAA-silver nanofibers could gradually turn darker is the generation of metallic Ag nanoparticles.

**Figure 3:**
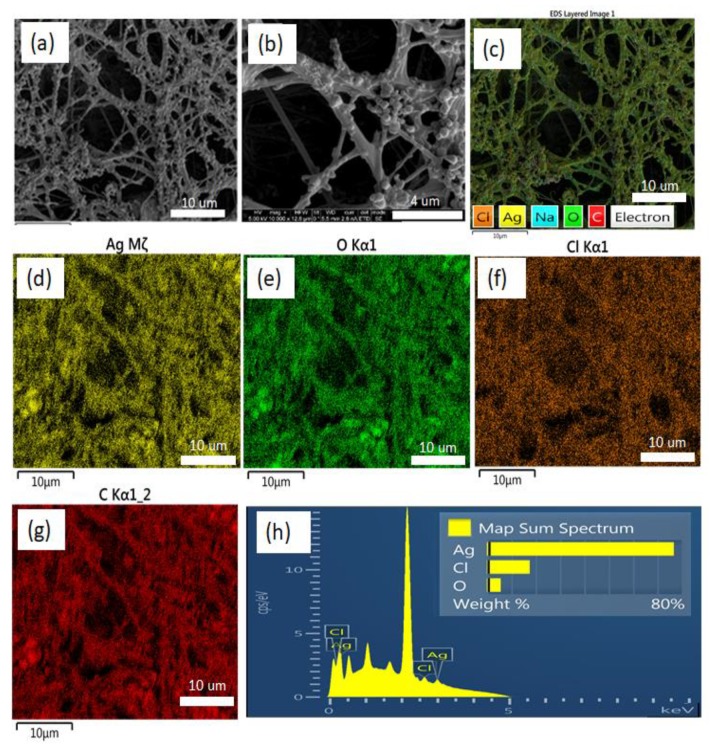
(**a**,**b**) Representative SEM micrographs of silver containing PAA nanofibers. EDS elemental maps from (**c**) all elements (**d**) Ag, (**e**) O, (**f**) Cl, (**g**) C on the PAA-silver nanofibers substrate. (**h**) is the EDS of the PAA-silver nanofibers.

Figure 3 shows the SEM images of PAA-silver electrospun fibers with high surface area, high uniformity, and homogeneity. The silver nanoclusters are clearly visible on the hydrogels. The silver clusters with a size of 0.5 to 1.5 µm were adhered to the PAA electrospun hydrogel due to the strong interaction between silver and carboxylic groups of PAA. 

In the EDS pattern, C, O, and Ag peaks are shown clearly demonstrating that hybrid PAA-silver materials were successfully prepared (Figure 3d–g). Other lines of the EDX spectrum are related to other elements in the material (mostly Na and Cl). The EDX analysis confirms the presence of silver within the hydrogel nanofibers and formation of nanoparticles on the surface of nanofibers, which is in good agreement with literature [24]. SEM/EDS analysis showed that the average size of silver particles was 290 nm. 

### 3.2. Optical Characterization 

**Figure 4:**
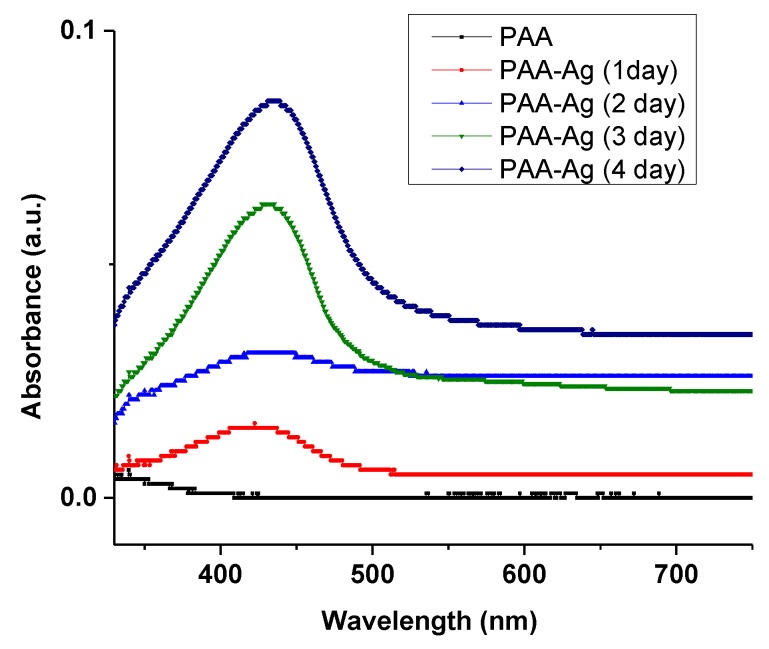
UV-vis spectra of silver nanoclusters growing in electrospun PAA–silver hydrogel (5 mM AgNO_3_) in respect to the time.

UV-visible absorption is an alternative method to confirm the formation of silver NPs in the electrospun PAA hydrogel. The UV-vis absorption spectra of pure PAA and silver NP-containing PAA fibers are demonstrated in Figure 4. The electrospun PAA did not show any peak in the region of 300 to 500 nm, while the PAA-silver hydrogel showed a strong absorption peak around 430 nm related to the cluster-polymer interactions [25,26]. This peak is due to the plasmon resonance excitations from the quantum size of silver NPs [27]. The increment of the absorption peak in the UV-vis represents the formation of NPs through chemical reduction process, which indicates the formation and growth of silver NPs over time. It is to be noted that the kinetics of silver loading was slow in the first and second days and followed by a rapid increase in the third and fourth days. 

### 3.3. XRD Study

**Figure 5:**
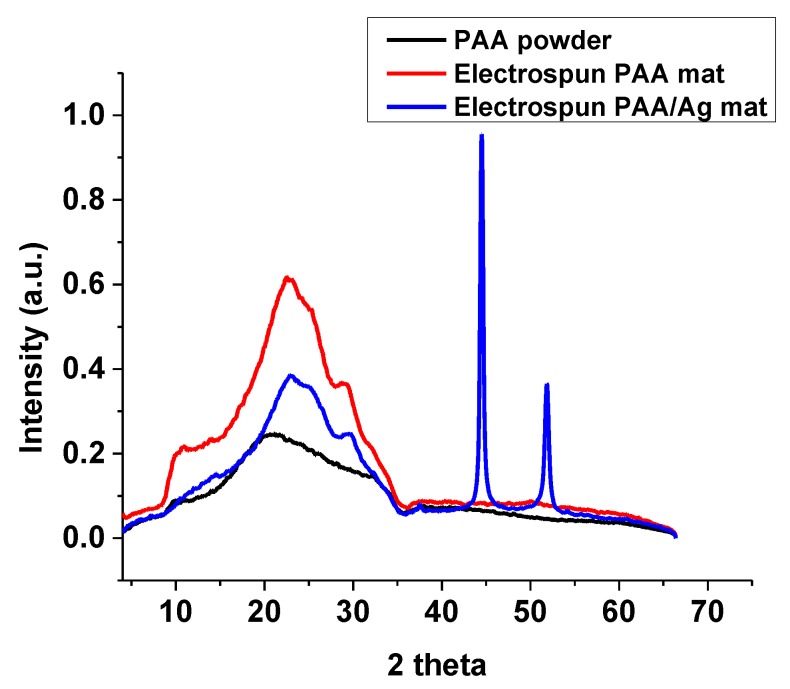
The XRD patterns of PAA powder, crosslinked electrospun PAA, and electrospun PAA-silver nanofibers (5 mM AgNO_3_).

The XRD pattern of silver NPs in PAA fibers is demonstrated in Figure 5. The XRD of crosslinked PAA-silver electrospun hydrogel exhibited two peaks at 44.52° and 51.82° that corresponded to the crystal faces of (200) and (111) planes of the face centered cubic (fcc) of silver NPs (Figure 5). Therefore, these results indicate metallic silver NP formation with high crystallinity. The XRD pattern revealed a lattice constant of 4.08620, which is in agreement with previously reported data (α = 4.0862) [28]. 

### 3.4. XPS Study

**Figure 6:**
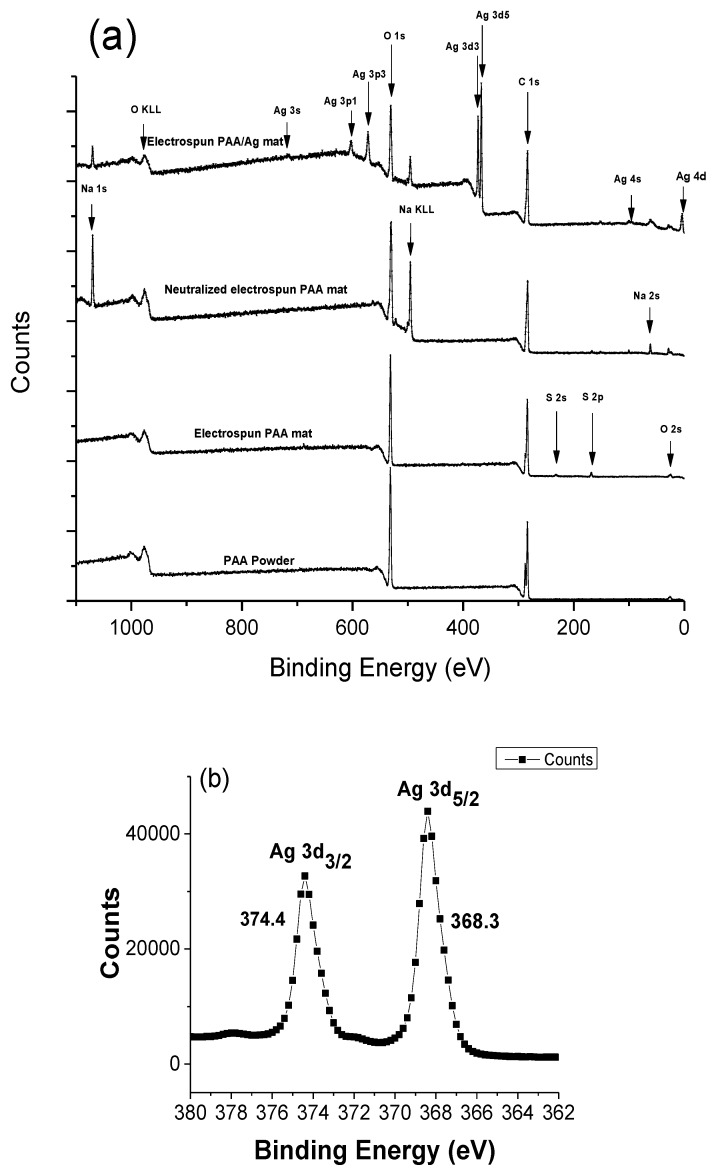
(**a**) XPS survey spectrum collected for the Ag NPs/PAA, and (**b**) High resolution XPS data of silver NPs loaded on the surface of electrospun nanofibers.

To confirm the formation of Ag NPs from the chemical reduction method, XPS was used to identify the change in the reduction process from the preparation of electrospun PAA to the reduction of silver ions using citrate ions. The upper most spectrum of Figure 6a shows the composition of the sample after Ag Np formation, revealing silver, oxygen, sodium, and small quantities of chlorine, and sulfur. The appearance of Cl, Na, and S comes from sodium citrate, sodium chloride, and sulfuric acid during both the neutralization and thermal esterification processes. The XPS analysis elucidated the surface state composition of the crosslinked PAA-silver materials. Figure 6b shows the full spectrum reveals silver to exist with two different binding energies.

Since metallic and oxidized silver have close characteristic state, the binding energy position of Ag3d could not be clearly identified by oxidation state of the Ag species [29,30]. Recent studies showed that the partially oxidized Ag NPs demonstrate better antibacterial activities than zero-valent Ag [31]. The atomic percentage of crosslinked PAA/Ag hydrogel are reported in Table 1.

High resolution XPS was used to detect the spectrum in the Ag 3d region (Figure 6b). Our results showed that the XPS spectra reveals two binding energy peaks at 374.4 eV and 368.3 related to 3d_3/2_ and Ag_5/2_, respectively. Compared to Ag^0^ (374.25 and 368.25 eV) and Ag_2_O (373.70 and 367.70 eV) [32], our results demonstrated that the observed shift of the peak position in respect to Ag^0^ is negligible. A tiny shift might be because of residual N, O, and C. These data confirmed that silver NPs were successfully loaded on the surface of electrospun nanofibers.

### 3.5. Antimicrobial Activity

**Figure 7:**
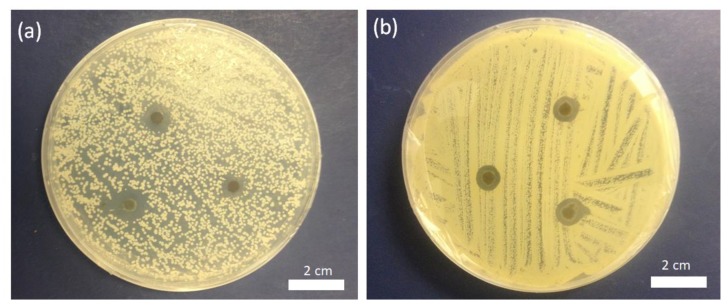
Zones of growth inhibition of (**a**) *C. albicans* fungi and (**b**) Methicillin-resistant *Staphylococcus aureus* (MRSA) bacteria for PAA-silver nanofibers soaked in 5 mM AgNO_3_ concentration. The antimicrobial activity of untreated PAA nanofibers was not shown here.

To further assess the feasibility of antimicrobial activities, we tested the antifungal and antibacterial activities of the embedded silver NPs in nanofiber scaffolds (Figure 7a,b). The results were obtained from the disc diffusion method against MRSA and *C. albicans*. On the other hand, the PAA-silver nanofiber scaffold produced zones of inhibition against *C. albicans*, and MRSA (Figure 7a,b) indicating the antimicrobial activity of incorporated Ag in PAA nanofiber scaffold soaked in 5 mM AgNO_3_ solution). The zone of inhibition of MRSA and *C. albicans* was found 3 ± 0.3 mm, and 2 ± 0.2 mm in the well treated with Ag NPs embedded PAA scaffolds, which showed maximum activity bacteria when compared with fungal strains. The zone of inhibition was measured as the maximum distance from the PAA-silver nanofiber scaffold in a number of directions at which an inhibition of bacterial growth was observed. This approach was already chosen in earlier studies [32,33]. These results showed that Silver NPs had multiple inhibitory actions for the interruption and blockage of activity against fungal and bacterial strains, which will minimize expenses required for disease control. 

However, the mechanisms responsible for the antimicrobial action of silver NPs remain unclear. Different mechanisms can lead to the action of Ag NPs against bacteria and fungi. According to Guzman et al. [34], (1) the inhibition of protein synthesis, (2) inhibition of a metabolic pathway, (3) interference with cell wall synthesis, and (4) interference with nucleic acid synthesis are the most common mechanisms of action of Ag NPs as antibacterial materials. Feng at al proposed three mechanisms of action responsible for the bacterial activity of Ag NPs [35]. In the first mechanism, Ag NPs penetrate to the cytoplasm and interact with the thiol and phosphorus compounds and therefore inactivate the replication of DNA or enzymes, which in turn affects the cell viability. In the second mechanism, Ag NPs attach to the surface of cell membranes, and thereby disturb the permeability and metabolic pathway of the cell wall and cause further cell death. Third, Ag NPs can also be oxidized in the presence of aqueous solutions to yield Ag^+^ ions. The Ag^+^ ions can interact with cytoplasmic components and nucleic acid and inactivate the cellular proteins and the replication ability of DNA.

## 4. Conclusions

In summary, we have demonstrated developed silver-PAA demonstrated fair antimicrobial activities against *C. albicans* (fungal agent) and MRSA (bacterial agent). The silver citrate complex has been found to be a promising substrate for selectivity incorporating silver nanostructures in PAA matrices. Therefore, the PAA-silver has a strong potential for antimicrobial application in preventing/treating infections. We conclude that silver NP patches exhibited multiple inhibitory actions against fungal and bacterial strains and has the potential as an antimicrobial agent in the infectious diseases. The silver embedding scaffolds represent a potentially attractive opportunity for clinical studies, such as biomedical labeling, wound healing, and cancer therapy. 

## Figures and Tables

**Table 1:** The atomic percentage measured for PAA-silver hydrogels soaked in 5 mM AgNO_3_ solution.

C 1s	O 1s	Ag 3d	Na 1s	Cl 2p	S 2p
63.2	27.4	5.4	3.5	0.3	0.3
